# Age-Related Increases in Adverse Seating and Mobility Events Among New Mobility Device Users

**DOI:** 10.1093/geroni/igaf122.3355

**Published:** 2025-12-31

**Authors:** Kevin Pritchard, Corey Morrow, Mariana Pacheco Busquets, Gede Pramana, Richard Schein, Mark Schmeler

**Affiliations:** Brigham and Women’s Hospital, Boston, Massachusetts, United States; Medical University of South Carolina, Charleston, South Carolina, United States; University of Pittsburgh, Pittsburgh, Pennsylvania, United States; University of Pittsburgh, Pittsburgh, Pennsylvania, United States; University of Pittsburgh, Pittsburgh, Pennsylvania, United States; University of Pittsburgh, Pittsburgh, Pennsylvania, United States

## Abstract

We described age-related increases in hospitalizations, pressure ulcers, or becoming stranded from a mechanical issue after receiving a new mobility device. This retrospective cohort study included 5,983 individuals who received new mobility devices from 87 U.S. clinics (48 states) using the Functional Mobility Assessment Registry from 2015-2024. Age was self-reported and dichotomized at ≥ 65 years old upon device delivery. Outcomes included self-reported hospitalization for a seating-related issue, pressure ulcer, or becoming stranded due to device malfunction. We estimated age-stratified rates of these outcomes using the Kaplan-Meier method. We used proportional hazards regression to assess the association between age and the number of days until each outcome, after controlling for 16 confounding factors like sociodemographic status, prior device use and experience, health status, physical function, and history of each outcome. Our sample was on average age 60 [SD = 18] years old, 47% were ≥65 years old, 54% women, and 26% were racial or ethnic minorities. The 365-day event rates for seating-related hospitalization, pressure ulcer, and stranding were 53.32% (95% CI: 51.59%-55.07%), 57.01% (55.28%-58.74%), and 56.34% (54.61%-58.08%) in the younger group and 55.33% (53.48%-57.19%), 58.38% (56.54%-60.23%), and 58.09% (56.25%-59.94%) in the older group. After multivariable adjustment, adverse events increased with age. Compared to adults under 65 years old, those 65 and older were more likely to have a seating-related hospitalization (HR: 1.12; 95% CI: 1.05-1.19), pressure ulcer (HR: 1.09; 95% CI: 1.03-1.16), or stranding (HR: 1.10; 95% CI: 1.04-1.17). Older age increased the likelihood of adverse events after receiving new mobility devices.

